# *Clostridium difficile* stool shedding in infants hospitalized in two neonatal intensive care units is lower than previous point prevalence estimates using molecular diagnostic methods

**DOI:** 10.1186/s12887-018-1113-z

**Published:** 2018-04-13

**Authors:** Andrea Green Hines, Alison Freifeld, Xing Zhao, Ann Anderson Berry, Lynne Willett, Peter C. Iwen, Kari A. Simonsen

**Affiliations:** 1 Adult Infectious Diseases, University of Nebraska Medical Center, Omaha, NE USA; 2 Pediatric Infectious Diseases, University of Nebraska Medical Center, Omaha, NE USA; 3 Neonatology, University of Nebraska Medical Center, Omaha, NE USA; 4 Pathology and Microbiology, University of Nebraska Medical Center, Omaha, NE USA

**Keywords:** Clostridium difficile, Infant, Epidemiology, Molecular epidemiology

## Abstract

**Background:**

The point prevalence of *Clostridium difficile* stool shedding in hospitalized infants from two neonatal intensive care units (NICUs) was examined utilizing standard clinical testing compared with duplex PCR to identify toxigenic and non-toxigenic *C. difficile* strains.

**Methods:**

All infants from the two NICUs affiliated with a single academic medical center were eligible for inclusion. Stool collection was blinded to patient characteristics and occurred during a one week period at each NICU and repeated with a second weeklong collection 6 months later to increase sample size. Stools were tested for *C. difficile* using EIA (GDH/toxin A/B) with samples testing +/+ or +/− subsequently evaluated by Loop-Mediated Isothermal Amplification (LAMP) and by duplex PCR amplification of *tcdB* and *tpi* (housekeeping) genes. Cytotoxicity assays were performed on all samples positive for *C. difficile* by any modality.

**Results:**

Eighty-four stools were collected from unique infants for evaluation. EIA results showed 6+/+ [7.1%], 7 +/− [8.3%], and 71 −/− [84.5%] samples. All 6 EIA +/+ were confirmed as toxigenic *C. difficile* by LAMP; 6/7 EIA +/− were negative by LAMP with one identified as invalid. Duplex PCR concurred with LAMP in all 6 stools positive for toxigenic *C. difficile*. PCR identified 2 EIA −/− stools positive for *tpi*, indicating shedding of non-toxigenic *C. difficile*. Cytotoxicity assay was positive in 4/6 duplex PCR positive samples and negative for all stools that were EIA +/− but negative by molecular testing.

**Conclusions:**

*C. difficile* blinded point prevalence in infants from two NICUs was 7.1% by molecular methods; and lower than expected based on historical incidence estimates. In house duplex PCR had excellent concordance with clinically available LAMP and EIA tests, and added detection of non-toxigenic *C. difficile* strain shedding. Evolving NICU care practices may be influencing the composition of infant gut microbiota and reducing the point prevalence of *C. difficile* shedding in NICU patient stools.

## Background

The epidemiology of *Clostridium difficile* infection (CDI) has shifted in the last decade and is now affecting populations previously at low risk to include healthy adults, peripartum women and young children [1]. Based on several recent studies, traditional risk factors for CDI, including antimicrobial exposure and recent hospitalization are absent in a major proportion of cases [2–4]. These epidemiological shifts in CDI have prompted renewed investigation into potential reservoirs and vectors for transmission. Asymptomatic shedders of *C. difficile*, including infants, have been suggested as playing a role [5–11]. A single-center study demonstrated that based on multilocus variable number of tandem repeats analysis (MLVA), 29% of hospital acquired CDI (HA-CDI) cases were highly related to *C. difficile* isolates from asymptomatic patients that were collected before the HA-CDI isolate [7]. A more recent investigation noted that asymptomatic *C. difficile* carriers increased the risk of nosocomial CDI in other hospitalized patients [12]. A wide range of asymptomatic colonization rates with toxigenic and non-toxigenic *C. difficile* have been reported in both hospitalized and community-dwelling infants from 11 to 71% [8, 10, 13–29]. NICU infants have been reported to have a prevalence of *C. difficile* colonization of between 15 and 78% based on several previously published studies performed in the U.S. and elsewhere [13, 18–20, 25, 30–42] (see Table 1). These studies also showed that confirmation testing using the cytotoxicity assay or PCR showed prevalence of toxigenic *C. difficile* to range from 0 to 67%.

**Table 1:** Prior NICU studies examining *C. difficile* prevalence

Author, Year of Study	Location	Test Methods	Prevalence of *C. difficile*
Kim, 1981 [37]	U.S.	Culture + cytotoxicity assay	21% culture +, 14% toxin +
Blakey, 1982 [31]	Australia	Culture	0–35% culture +^a^
Donta, 1982 [18]	U.S.	Cytotoxicity assay	54.9% toxin +
Sherertz, 1982 [25]	U.S.	Culture	59% culture +
Malamou-Ladas, 1983 [39]	England	Culture	54% culture +
Al-Jumaili, 1984 [13]	England	Culture + cytotoxicity assay	71% culture +, 45% toxin +
Lishman, 1984 [38]	England	Culture + cytotoxicity assay	78% culture +, 67% toxin +
Phua, 1984 [40]	England	Culture + cytotoxicity assay	21% culture +, 0% toxin +
Zedd, 1984 [42]	U.S.	Culture	41% culture +
Cardines, 1988 [32]	Italy	Culture + cytotoxicity assay + PAGE^b^	63% culture +, 0% toxin + (per cytotoxicity assay), 16% toxigenic strain + (per PAGE)
el-Mohandes, 1993 [34]	U.S.	Culture + cytotoxicity assay	15–33% culture +, 71–100% toxin +^c^
Kato, 1994 [36]	Japan	Culture + PCR for toxins A and B	61% culture +, 6% toxin +^d^
Tina, 1994 [41]	Italy	Culture + EIA for toxins A and B	43.6% culture +, 31.2% toxin +
Enad, 1997 [19]	U.S.	EIA for toxin A	52% EIA +
Alfa, 2002 [30]	Canada	PCR for *C. difficile* 16S gene	21% *C. difficile* 16S gene +
Chang, 2012 [33]	Korea	PCR for *C. difficile* 16S gene + PCR for toxins A and B	34.7–53.1% *C. difficile* 16S gene+^e^ 23.5–30.8% toxin +
Ferraris, 2012 [35]	France	PCR for *C. difficile* 16S gene	42.1% *C. difficile* 16S gene+
Faden, 2015 [20]	U.S.	EIA GDH Ag/toxins A/B *C. difficile* culture	25.7% +^f^

^a^Study measured prevalence at days 0–4, 5–8, 9–12, 13–16, 17–20 and > 20 days, thus providing a prevalence range

^b^SDS-polyacrylamide gel electrophoresis (PAGE) of EDTA-extracted proteins used to identify toxigenic strains

^c^Study measured prevalence after 1 week of enteral feeding, at 15 +/− 1 days of life; 2 more specimens were collected at 2 week intervals, 24 +/− 1 and 32 +/− 2 days of life, thus providing a prevalence range

^d^PCR for toxins A and B were performed on only 32 of 41 *C. difficile* culture+ infants

^e^Study measured prevalence within 72 h of birth, 1, 2, and 4–6 weeks of age thus providing a prevalence range

^f^Test modality of positivity unspecified

During the last three decades substantial advances in NICU care have occurred. These include earlier feeding, emphasis on human milk feedings, use of more broad-spectrum antibiotics as well as additional efforts to control antimicrobial exposure through stewardship, and the survival of very low birth weight infants with prolonged, complicated hospital stays. Despite these important medical practice changes and the evolution of more precise molecular laboratory tests for toxigenic *C. difficile*, the prevalence of *C. difficile* has not been re-evaluated in U.S. NICU settings with molecular technology.

We examined the current point prevalence of *C. difficile* stool shedding in hospitalized infants from two affiliated NICUs utilizing a rapid and novel duplex PCR which was developed and validated in our laboratory [43]. This duplex PCR detected the presence of two genes, (*tpi* and *tcdB*) and we proposed the NICU as a high prevalence unit for validation of the PCR method.

All *C. difficile* strains, toxigenic and non-toxigenic, possess the housekeeping gene *tpi* (triose phosphatase isomerase). The *tcdB* gene encodes for the *C. difficile* toxin B. A non-toxigenic strain was defined as the detection of the *tpi* gene alone while a toxigenic strain was defined as the detection of both *tpi* and *tcdB* genes. We hypothesized that with both the epidemiologic changes in CDI as well as the advances in NICU care, the prevalence of *C. difficile* stool shedding may be rising, and heighten concerns for risk to hospital patients and the hospital environment.

## Methods

All infants hospitalized in two NICUs (NICU A and B) producing stool during the study period were included in the point prevalence survey. The institutional IRB reviewed and approved the protocol and waived informed consent as no patient identifiers were maintained for the study.

Collection of the stool samples occurred over two separate weeks in each NICU. At NICU A, stool samples were collected in March and in September; at NICU B, stool samples were collected in April and in September. NICU A is a Level III, 36-bed NICU and NICU B is a Level IV, 42-bed NICU. At the beginning of each study week and at the time of any new NICU admission during the study week, five sticker labels containing unique study numbers were placed at the bedside of each NICU patient. Each NICU bedside nurse collected patient’s stool soiled diapers, placed the diaper in a sealed container labeled with the study number and date and subsequently deposited the specimen container into a specially labeled bin located in the NICU. Nurses were instructed to collect up to five stool soiled diapers per patient. This collection methodology ensured patient non-duplication at the clinical level with blinding of the study team. Each NICU had a neonatologist on the study team who was also able to ensure non-duplication and who did not have access to the stool results on a per patient level. A study researcher collected the stool samples from the bin periodically each day and transported them to the research laboratory.

**Fig. 1:**
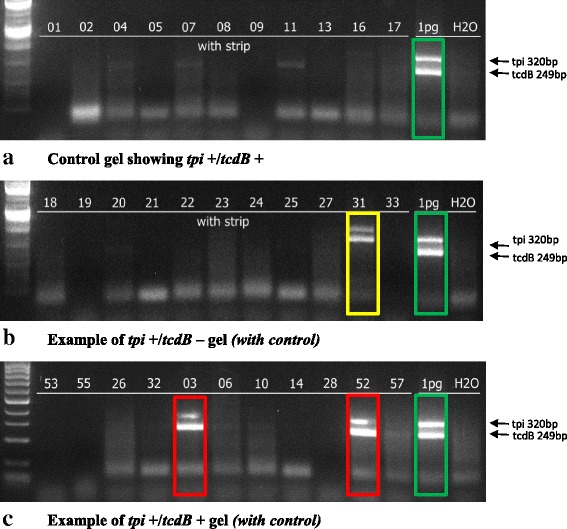
Gel electrophoresis examples for *C. difficile tpi* and *tcdB* results reporting. **a**. Control gel showing *tpi* +/*tcdB* +. **b**. Example of *tpi* +/*tcdB* – gel *(with control).* **c**. Example of *tpi* +/*tcdB* + gel *(with control)*

Stool from each NICU patient’s soiled diapers was divided into five 1 mL aliquots and frozen at − 20^0^ C until DNA extraction and PCR testing. The procedure is briefly described: DNA extraction was performed with liquid stool combined with lysis reagents and processed in a 1 ml-capacity lysis microreactor (LMR) which employed intense mixing with heating resulting in bacterial cell lysis. A surface-treated polystyrene strip bound DNA released by lysis from the mixture and permitted transfer of the DNA on the strip to the PCR cuvette. A rapid thermocycler (Philisa Thermal Cycler, Streck, Inc., Omaha, NE) was used to specifically amplify a conserved region of both toxigenic and non-toxigenic *C. difficile tpi* gene and a non-repeat region of the toxigenic *C. difficile tcdB* gene using primers designed using online multiplex PCR primer design software called “Primo Multiplex 3.4”. (http://www.changbioscience.com/primo/primoml.html). The forward *tpi* gene primer was TATATGTGCACCATTTACTTTATT and the *tpi* reverse primer was AACTTTACAAACATCTTTAGTTTTT, generating a 320 bp PCR product. The forward *tcdB* gene primer was TTAGCAGGAATTTCAGCAGGT and the reverse *tcdB* gene primer was ATGACCTGAACCACCTTCCA, generating a 249 bp product. Each 25 μl reaction contained a final concentration of 0.2 mM dNTPs, 5.5 mM MgSO4, 0.5 U KOD Hot Start DNA polymerase, 1X PCR buffer (PCR kit, EMD Chemicals, Inc), 0.4 mg/ml BSA (Ambion, Inc), 0.2 μM forward and 0.2 μM reverse primers (Sigma-Aldrich, St. Louis, MO). Amplification was completed in 19 min as more fully described in a previous study [43]. The thermal protocol included an enzyme activation step at 95 °C for 30 s followed by 45 cycles of 95 °C for 6 s and 56 °C for 6 s, and 72 °C for 6 s. Gel electrophoresis was utilized for identifying bands corresponding to the molecular weights for *tpi* and *tcdB* amplified fragments (Fig. 1).

All stool samples were additionally tested for *C. difficile* antigen glutamate dehydrogenase (GDH and *C. difficile* toxins A and/or B by enzyme immunoassay (EIA) (*C. diff* Quik Chek Complete, Alere Inc., Waltham, MA). Samples that were discordant (+/−) or positive/positive (+/+) for GDH and toxins A/B had reflex testing using LAMP technology (illumigene®, Meridian Bioscience, Inc., Cincinnati, OH). These commercially available tests were performed by the hospital clinical laboratory for comparison of the research method to current standard of care clinical tests. Cytotoxicity assays were performed on all samples positive by any modality. The *Clostridium difficile* Toxin/Antitoxin kit (TechLab, Blacksburg, VA) was used for the detection of *C. difficile* toxin in stool specimens by following manufacturer’s instructions. Specimens that showed characteristic cytotoxin activity after inoculation of MRC-5 tissue culture cells (rounding of the cells) which were neutralized by *C. difficile* antitoxin were considered positive for *C. difficile* toxin.

## Results

**Fig. 2:**
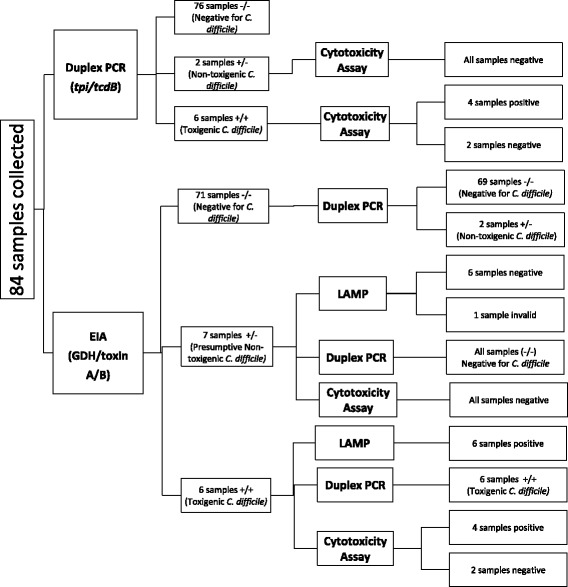
Pathway for testing of stool samples collected from NICU babies using duplex PCR and standard clinical lab methods for the detection of *Clostridium difficile*

Eighty-four stool samples from unique infants were collected during the study (Fig. 2). The number of samples collected from each NICU was unknown as no patient identifiers were maintained with the specimens. Seventy-one samples were EIA −/−, 7 samples were EIA +/− and 6 were EIA +/+. All 6 EIA +/+ samples were confirmed as toxigenic *C. difficile* by LAMP technology and also concurred with the results of the in house duplex PCR. Therefore, the point prevalence of toxigenic *C. difficile* in our NICU population was 7.1% (6/84). Cytotoxicity assay was performed on positive samples (by any test) for additional confirmation and was positive for 4/6 duplex PCR samples that were positive for toxigenic *C. difficile*; 2 samples could not be confirmed. Six of the 7 EIA +/− samples were negative for toxigenic *C. difficile* by LAMP technology and one sample was invalid. Our duplex PCR and the cytotoxicity assay were negative for all 7 of these samples. Our duplex PCR was negative for 69 of the 71 EIA −/− samples. The other 2 EIA −/− samples were positive for the *tpi* gene but negative for *tcdB*, indicating non-toxigenic *C. difficile*. Thus, the overall point prevalence of toxigenic (*n* = 6) and non-toxigenic (*n* = 2) *C. difficile* shedding in our NICU population was 9.5% (8/84) (Table 2).

**Table 2:** NICU stool samples positive for *C. difficile* by one or more modalities

Number of samples (n)	EIA GDH/toxin A/B	LAMP technology	Duplex PCR (*tpi*/*tcdB*)	Cytotoxicity assay
2	−/− Negative	Not done	+/− Nontoxigenic *C. difficile*	Negative
4	+/+ Toxigenic *C. difficile*	Positive	+/+ Toxigenic *C. difficile*	Positive
2	+/+ Toxigenic *C. difficile*	Positive	+/+ Toxigenic *C. difficile*	Negative
7	+/− Presumptive Nontoxigenic *C. difficile*	Negative^a^	−/− Negative	Negative

^a^one sample specimen was invalid

## Discussion

The point prevalence of *C. difficile* stool shedding in hospitalized infants from two affiliated NICUs was examined utilizing standard clinical testing (EIA with reflexive molecular identification via LAMP) and an in house duplex PCR that identified toxigenic and non-toxigenic *C. difficile* strains. We hypothesized that the prevalence of NICU *C. difficile* shedding would be higher than previous reports in part due to increased sensitivity of molecular testing compared to the testing modalities used in most previous studies (culture, cytotoxicity assay and EIA). The increased sensitivity of molecular testing contributing to the increase in *C. difficile* prevalence has been observed in previous studies [44–47]. Additionally, we surmised that the epidemiologic changes in CDI as well as the advances in NICU care would contribute to a higher prevalence of *C. difficile* shedding in NICUs over time. However, on the contrary, we demonstrated a prevalence of 7.1% for toxigenic *C. difficile* and 9.5% for both toxigenic and non-toxigenic *C. difficile* strains, which is substantially less than previously published reports suggesting a mean prevalence of at least 21% in the NICU population [13, 18–20, 25, 30–42]. Our secondary aim was achieved in that our stool lysis technique and rapid duplex PCR had excellent concordance with commercial EIA and LAMP testing, and moderately good concordance with cytotoxicity tests. This suggests that the duplex PCR could be used more broadly for rapid, accurate clinical diagnosis and for further epidemiologic studies of *C. difficile* stool shedding with and without toxin production. Stool isolates testing +/− on EIA but negative by both LAMP and duplex PCR may have been from GDH cross-reactivity with other organisms [48, 49].

The identified difference between the prevalence of NICU *C. difficile* shedding in our study and previous studies may still be a reflection of advances in NICU practices. One important practice change is the emphasis of using human milk for feedings, perhaps decreasing the colonization of *C. difficile* in the infant gut [17, 22, 50, 51]. Another hypothesis for this change is the evolution of infection control measures with greater emphasis on caregiver hand hygiene and a transition from open ward NICUs to private patient rooms. These improvements in infection control within a NICU could decrease the transmission and thus prevalence of *C. difficile* shedding in the NICU environment.

Our study had several limitations. As no patient identifiers were maintained with the stool specimens, we were unable to obtain any clinical data on the infants. We were therefore unable to investigate possible clinical correlates with *C. difficile* shedding in these NICU infants. We did not test for the B1/NAP1/027 strain, which may contribute to increased incidence and severity of CDI, since we found a low prevalence of NICU *C. difficile*. Additionally, the use of previously frozen stool specimens may have impacted the sensitivities of the tests.

Additional NICU-based studies examining the clinical correlations of infant *C. difficile* colonization and shedding are needed to further answer questions regarding the epidemiologic changes in CDI. A lower point prevalence of NICU *C. difficile* as defined by our study is meaningful in that it informs sample size calculation for future work of clinical correlates of asymptomatic *C. difficile* colonization in the NICU. Potential future directions in NICU *C. difficile* colonization and shedding research include a follow-up survey of NICU infants with specific attention to mode of delivery, use of antibiotics, timing of initial feeding and number of hospitalization days. Additionally, as the epidemiology of CDI evolves, studies are needed to evaluate the potential for colonized NICU infants to serve as a reservoir or vector for transmission of toxigenic *C. difficile* strains to healthcare workers, the hospital environment, and vulnerable populations within and outside the hospital.

## Abbreviations

CDI: *Clostridium difficile* infection
EIA: Enzyme immunoassay
GDH: Glutamate dehydrogenase
HA-CDI: Hospital acquired *Clostridium difficile* infection
LAMP: Loop-mediated isothermal amplification
LMR: Lysis microreactor
MLVA: Multilocus variable number of tandem repeats analysis
NICU: Neonatal Intensive Care Unit
